# Health extension service utilization in Ethiopia: systematic review and meta-analysis

**DOI:** 10.1186/s12913-024-11038-4

**Published:** 2024-04-26

**Authors:** Misganaw Guadie Tiruneh, Eneyew Talie Fenta, Destaw Endeshaw, Amare Mebrat Delie, Ousman Adal, Abiyu Abadi Tareke, Eyob Ketema Bogale, Tadele Fentabel Anagaw

**Affiliations:** 1 Department of Health Systems and Policy, Institute of Public Health, College of Medicine and Health Sciences, University of Gondar, P.O. Box: 196, Gondar, Ethiopia; 2 Department of Public Health, College of Medicine and Health Sciences, Injibara University, Injibara, Ethiopia; 3 Department of Adult Health Nursing, School of Health Sciences, College of Medicine and Health Sciences, Bahir Dar University, Bahir Dar, Ethiopia; 4 Department of Emergency and Critical Care Nursing, College of Medicine and Health Sciences, Bahir Dar University, Bahir Dar, Ethiopia; 5 Amref Health Africa in Ethiopia, SLL project COVID-19/EPI technical assistant at West Gondar Zonal Health Department, Gondar, Ethiopia; 6 Department of Health Promotion and Behavioral Sciences, School of Public Health, College of Medicine and Health Sciences, Bahir Dar University, PO. Box.079, Bahir Dar, Ethiopia

**Keywords:** Health extension services, Utilization, Ethiopia, Meta-analysis

## Abstract

**Introduction:**

Ethiopia strives to achieve Universal Health Coverage (UHC) through Primary Health Care (PHC) by expanding access to services and improving the quality and equitable comprehensive health services at all levels. The Health Extension Program (HEP) is an innovative strategy to deliver primary healthcare services in Ethiopia and is designed to provide basic healthcare to approximately 5000 people through a health post (HP) at the grassroots level. Thus, this review aimed to assess the magnitude of health extension service utilization in Ethiopia.

**Methods:**

The Preferred Reporting Items for Systematic Reviews and Meta-Analysis (PRISMA) checklist guideline was used for this review and meta-analysis. The electronic databases (PubMed, Cochrane Library, and African Journals Online) and search engines (Google Scholar and Grey literature) were searched to retrieve articles by using keywords. The Joanna Briggs Institute (JBI) meta-analysis of statistics assessment and review instrument was used to assess the quality of the studies. Heterogeneity was assessed using the I^2^ statistic. The meta-analysis with a 95% confidence interval using STATA 17 software was computed to present the pooled utilization of health extension services. Publication bias was assessed by visually inspecting the funnel plot and statistical tests using Egger’s and Begg’s tests.

**Result:**

22 studies were included in the systematic review with a total of 28,171 participants, and 8 studies were included in the meta-analysis. The overall pooled magnitude of health extension service utilization was 58.5% (95% CI: 40.53, 76.48%). In the sub-group analysis, the highest pooled proportion of health extension service utilization was 60.42% (28.07, 92.77%) in the mixed study design, and in studies published after 2018, 59.38% (36.42, 82.33%). All studies were found to be within the confidence interval of the pooled proportion of health extension service utilization in leave-out sensitivity analysis.

**Conclusions:**

The utilization of health extension services was found to be low compared to the national recommendation. Therefore, policymakers and health planners should come up with a wide variety of health extension service utilization strategies to achieve universal health coverage through the primary health care.

**Supplementary Information:**

The online version contains supplementary material available at 10.1186/s12913-024-11038-4.

## Introduction

Ethiopia strives to achieve Universal Health Coverage (UHC) through Primary Health Care (PHC) by expanding access to services and improving the provision of quality and equitable comprehensive health services at all levels [1, 2]. Primary Health Care (PHC) services are fundamental to improving health and health equity, particularly in the context of low-and middle-income countries [3, 4]. The Health Extension Program (HEP) is an innovative strategy to deliver PHC services in Ethiopia and provides a model for countries struggling to improve health outcomes in a resource-constrained setting [5, 6].

Ethiopia has been implementing a Community Health Program (CHP) called the Health Extension Program (HEP) since 2003, which is planned to improve the health of the community by focusing on preventive, promotional, and selected curative health services with a special focus on maternal and child health [7, 8], and it has made significant contributions in improving access and coverage of key primary healthcare services for the last 15 years [9, 10]. The HEP is intended to give ownership and responsibility for maintaining health to households so that communities are empowered to produce and maintain their health. The program involves women in decision-making processes and promotes community ownership, empowerment, autonomy, and self-reliance [9].

Health Extension Workers (HEWs) are responsible for implementing the 16 HEP packages in five categories. The five categories of HEP were family health services, personal hygiene and environmental sanitation, major communicable and neglected tropical diseases (NTDs), non-communicable diseases (NCDs), and health education and communication. The packages were considered relevant by policymakers to the rural communities and have been delivered through outreach, home visits, and static approaches [2, 11, 12]. The program expanded to urban centers with additional curative services in 2009 [9, 13]. Additionally, in 2016, the government added two new service packages that made up a total of 18 packages with additional standards of commodities and training of HEWs [9]. It is designed to provide basic healthcare to approximately 5000 people through a health post (HP) at the grassroots level. Every HP is staffed by two female health extension workers (HEWs) trained for one year and paid directly by the government [12]. The HEWs spend 75% of their time on home visits to teach and demonstrate HEP packages to family households and the rest of their time in the health post to provide basic health services [14, 15].

The primary purpose of the HEP is to improve access to and utilization of health care particularly for children and mothers [9, 16, 17]. Health service utilization is a result of multiple factors, such as health workers’ behavior and the characteristics of the community (family characteristics, social structure, and perceptions about modern health services) [15, 18]. It is also influenced by enabling factors, such as the availability of health facilities, accessibility to health services, quality of services, and affordability as well as the characteristics of complaints and the intensity of illness [15]. According to Andersen, factors associated with the utilization of health services can be categorized into predisposing, enabling, and needs factors [19].

According to the Ethiopian Demographic and Health Survey (EDHS) of 2016, the four consecutive EDHS starting in 2000, showed institutional delivery increased from 5 to 26%, antenatal care increased from 27 to 62%, modern contraceptive utilization increased from 6 to 35%, and infant mortality decreased from 97 to 48% [20]. Despite an encouraging trend of accomplishments, Ethiopia still has several poor health outcome indicators related to the health extension program [8]. The second Health Sector Transformation Plan (HSTP II) revealed that the proportion of model households is 18%, open defecation-free is 40%, and households having hand washing facilities with soap and water are 8%, while the targets in 2024/25 are 50%, 60%, 80%, and 58%, respectively [1], which needs an extensive effort in utilization of the health extension services to achieve the target. These poor outcomes are mainly due to low utilization of the HEP and poor access to health services [3, 4, 8].

In Ethiopia, different studies showed that different levels of health extension service utilization ranged from 9.3 to 86% [3, 4, 7, 8, 16, 21–27]. However, there is still no consistent evidence about the utilization of health extension services in Ethiopia. Therefore, this study was aimed to assess the utilization of health extension services in Ethiopia through a systematic review and meta-analysis. The findings of the study will contribute to the development of targeted strategies for the provision of health extension services, for designing public health interventions to improve the utilization of health extension services, and for strengthening the community HEP for UHC through primary health care services.

Research question: What is the pooled magnitude of health extension service utilization in Ethiopia?

## Methods and materials

### Information source and search strategy

This systematic review and meta-analysis was performed by following the Preferred Reporting Items for Systematic Reviews and Meta-Analyses (PRISMA) guidelines [28]. An electronic search strategy was implemented using databases (PubMed, Cochrane Library, and African Journals Online), which were systematically searched to retrieve related articles using keywords. Google Scholar and relevant grey literature were also searched. The literature search technique was conducted by using the keywords (“Service”) OR (“Package”) AND (“Utilization”) OR (“Uptake”) OR (“Usage”) AND (“Health extension worker”) OR (“Community health worker”) AND (“Ethiopia”) OR (“Ethiopian”). All studies conducted up to October 31/2023 were included. We also performed a manual hand search for reference lists of the articles found through the database search and included the articles relevant to our topic of review. The protocol for this systematic review and meta-analysis is registered in the international Prospective Register of Systematic Reviews (PROSPERO) and obtained a registration number of CRD 42,023,441,568.

### Eligibility criteria

For the review, the CoCoPop mnemonic (Condition, Context, and Population) was used to construct a clear and meaningful review question. Condition: health extension service utilization; Context: Ethiopia and Population: all people live in Ethiopia. Studies that reported the magnitude of health extension service utilization in Ethiopia using an observational study design (cross-sectional, case-control, and cohort) on the health extension services, with open or free access to full text and written in English were included.Studies without abstracts and full-texts, reports, and qualitative studies were excluded. Editorials, newspaper articles, and other forms of popular media reports were excluded, as were studies that did not report the magnitude of service utilization provided by health extension workers. Articles were assessed for inclusion using their title and abstract, and then a full review of the articles was done before they were included in the final review.

### Data extraction and management

Eligible studies were imported to Endnote v.20, and duplicates were removed. The four independent reviewers (MGT, ETF, DE, and AMD) did the abstract and full-text reviews and extracted data by the Microsoft Excel spreadsheet using a standardized data extraction checklist. Any disagreements and uncertainties during the extraction process were resolved through logical consensus among the four authors, and the final consensus was approved with the participation of the author (TFA). The following data were extracted: author, publication year, study design, place of study, type of package, sample size, and magnitude of service utilization.

### Quality assessment

The Joanna Briggs Institute (JBI) critical appraisal checklist was used to assess the quality of studies, which is freely available at https://jbi.global/critical-appraisal-tools. We used the following items to evaluate the studies: Inclusion criteria; Description of study subject and setting; Valid and reliable measurement of exposure; Objective and standard criteria used; Identification of confounders; Strategies to handle confounders; Valid and reliable measurement of outcome; and Appropriate statistical analysis. Using the tool as a protocol, the reviewers (MGT, OA, AAT, and EKB) evaluate the quality of the original articles independently. Those studies, with scores of 5 or more in the JBI critical appraisal were considered to have good quality and included in the review. Discrepancies in the quality assessment were resolved through the involvement of the author (TFA) (Table 1).

**Table 1:** Methodological quality assessment of included studies using the JBI critical appraisal checklist

Study	Inclusion in the sample clearly defined	Study subjects and the setting described in detail	Exposure measured in a valid and reliable way	Objective, standard criteria for measurement of the condition?	Confounding factors identified	Strategies to deal with confounding factors stated	Outcomes measured in a valid and reliable way	Was appropriate statistical analysis used?	Total score
Aynalem et al.	Y	Y	Y	Y	Y	Y	U	Y	7
Molla et al.	Y	Y	Y	Y	Y	Y	Y	Y	8
Girmay et al.	Y	Y	Y	Y	Y	Y	U	Y	7
Gebreegziabher et al.	Y	Y	Y	Y	Y	N	U	N	5
Sinki et al.	Y	Y	Y	Y	Y	Y	U	Y	7
Kelebessa et al.	N	Y	Y	Y	Y	Y	U	Y	6
Zeleke et al.	Y	Y	Y	Y	Y	Y	U	Y	7
Jikamo et al.	Y	Y	Y	N	Y	Y	N	Y	6
Gebretsadik et al.	Y	Y	Y	Y	Y	Y	Y	Y	8
Shaw et al.	N	Y	Y	Y	Y	Y	Y	Y	8
Berri et al.	Y	Y	Y	Y	Y	Y	Y	Y	8
Asmamaw et al.	Y	Y	Y	Y	Y	Y	Y	Y	8
Bayou et al.	N	Y	Y	Y	Y	Y	Y	Y	7
Jisso et al.	N	Y	Y	Y	Y	Y	Y	Y	7
Getnet et al.	Y	Y	Y	Y	Y	Y	Y	Y	8
Tesfau et al.	N	Y	Y	Y	Y	Y	Y	Y	7
Hadro et al.	N	Y	Y	N	Y	Y	Y	Y	6
Gebretsadik et al.	N	Y	Y	Y	Y	Y	U	Y	6
Birhanu et al.	N	Y	Y	Y	Y	Y	U	Y	6
Nigussie & Girma	N	Y	Y	Y	Y	Y	Y	Y	7
Gela et al.	N	Y	Y	N	Y	Y	U	Y	5
Yitayal et al.	Y	Y	Y	Y	Y	Y	Y	Y	8

### Statistical analysis

The data were extracted from the studies using Microsoft Excel V.2016, and the extracted data were exported to STATA-17 software for analysis. The articles were summarized by tables and forest plots. The standard error (SE) of health extension service utilization was calculated. The I^2^ statistical test was computed to check heterogeneity across the studies [29]. Since significant heterogeneity was detected across the studies, a meta-analysis using a random effects model was employed to estimate the pooled magnitude with a 95% CI. The presence of publication bias was checked visually by using a funnel plot and statistically by using Egger’s and Begg’s statistical tests [30]. Subgroup analysis was also done based on the study design and publication year. Sensitivity analysis was also done to evaluate the effect of each study on the pooled magnitude of health extension service utilization by excluding each study.

## Result

**Fig. 1:**
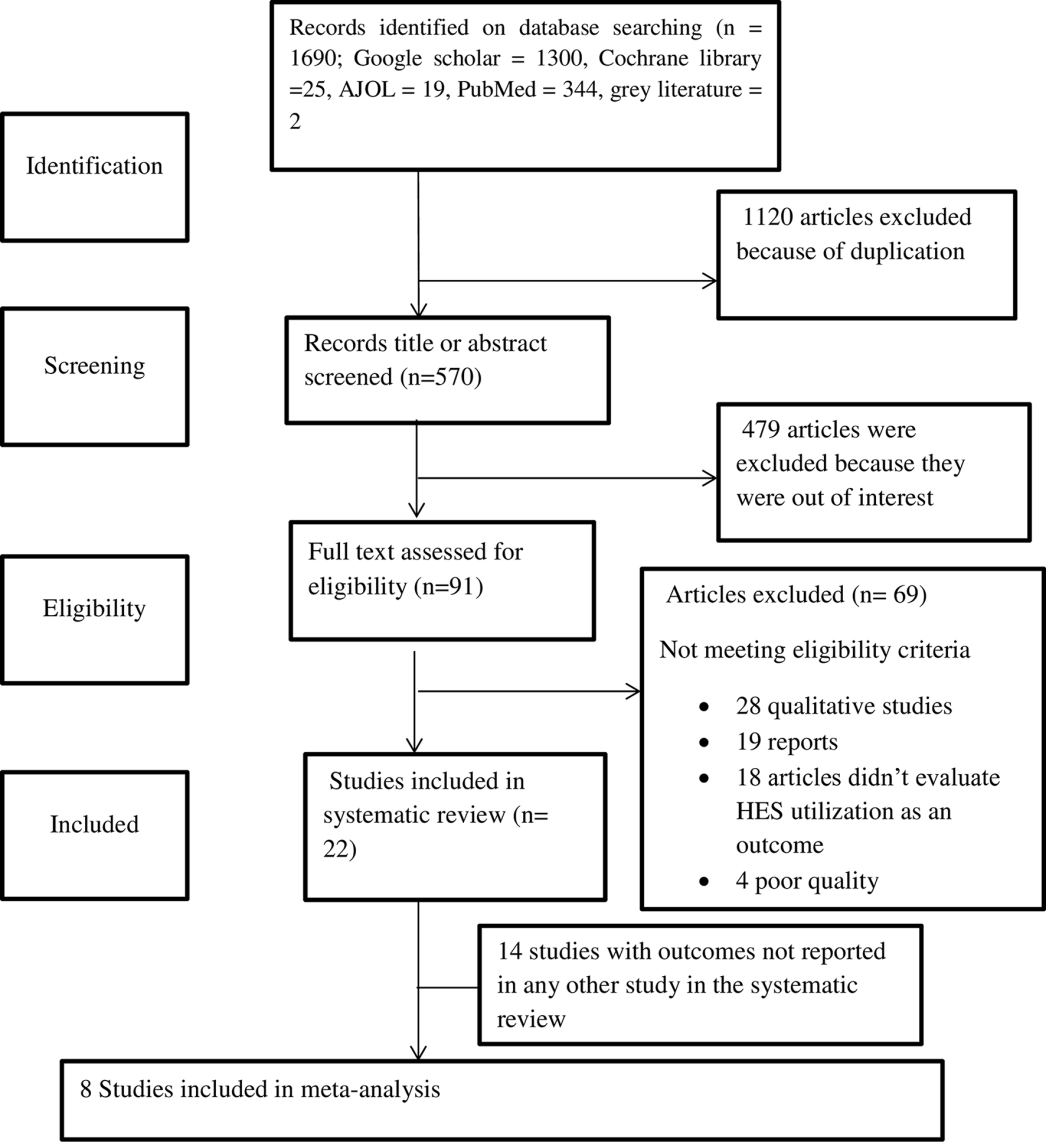
PRISMA flow diagram of study selection of health extension service utilization

A total of 1690 articles were identified through our initial database search. After duplicate records were removed, 570 records were reviewed by title and abstract. Ninety-one articles were included for full text review. Finally, twenty-two studies were included in the review after applying inclusion and exclusion criteria (Fig. 1). No additional studies were obtained after manual retrieval of the references of the included articles.

### Study characteristics

In the systematic review, 22 articles with 28,171 participants were included [3, 4, 7, 8, 16, 17, 21–27, 31–39]. Eight of the studies were from Oromiya [4, 7, 16, 23, 27, 37–39], six in the SNNP region [17, 22, 25, 31, 32, 34], four in the Amhara region [8, 21, 33, 36] and four of the articles were from Tigray, Somali, Addis Ababa, and nationwide each region accounts for one study [3, 24, 26, 35]. Fifteen studies were cross-sectional [3, 4, 8, 17, 22, 23, 26, 27, 32–38], and seven articles were mixed cross-sectional by study design [7, 16, 21, 24, 25, 31, 39]. The maximum sample size was 12,000 households in the Oromiya region [27] and the minimum sample size was 226 in the SNNP region [22]. The year of publication was ranged from 2013 to 2023, five studies were conducted in 2020, 14 studies were done between 2013 and 2019, and three studies were between 2022 and 2023. Five of the included studies [3, 7, 8, 16, 25] were assessed the utilization of urban health extension packages (Table 2). Eight studies reported the proportion of health extension service utilization without specification of the packages. From the included 22 studies, 14 studies reported different outcomes related to the utilization of health extension services, which weren’t reported in other studies. Out of 14 articles with different reported service outcomes, eleven studies [16, 17, 22, 23, 27, 31–35, 37] focused on the utilization of the maternal and child health service package of the health extension program. One of the studies in Somali region emphasized on tuberculosis screening service provided by HEWs [24], the others were aimed on the sexual reproductive health [26] and basic health services provided by HEWs [36].

**Table 2:** Characteristics of the studies included in the systematic review and meta-analysis of health extension service utilization in Ethiopia

S.no	Author	Publication year	Study design	Place of study	Type of package	Number of participants	Magnitude of service utilization
1	Girmay et al. [3]	2019	CS	AA	UHEP	628	86%
2	Molla et al. [8]	2020	CS	Amhara	UHEP	626	59.50%
3	Yitayal et al. [36]	2014	CS	Amhara	HEP	1318	78.50%
4	Kelbessa et al. [4]	2014	CS	Oromiya	HEP	806	39%
5	Bayou et al. [22]	2013	CS	SNNP	HEP	226	19%
6	Gela et al. [23]	2014	CS	Oromiya	HEP	703	73.10%
7	Shaw et al. [27]	2015	CS	Oromiya	HEP	12,000	9.30%
8	Jisso et al. [26]	2022	CS	Nationwide	HEP	902	19.18%
9	Asmamaw et al. [33]	2023	CS	Amhara	HEP	760	15.13%
10	Aynalem et al. [21]	2020	Mixed	Amhara	HEP	806	14.80%
11	Berri et al. [16]	2020	Mixed	Oromiya	UHEP	401	14.20%
12	Gebreegziabher et al. [7]	2017	Mixed	Oromiya	UHEP	418	72.80%
13	Getnet et al. [24]	2017	Mixed	Somali	HEP	380	20.30%
14	Negussie & Girma [17]	2017	CS	SNNP	HEP	613	69.82%
15	Jikamo et al. [25]	2019	Mixed	SNNP	UHEP	403	61.70%
16	Gebretsadik et al. [32]	2018	CS	SNNP	HEP	2040	12.40%
17	Hadro et al. [31]	2022	Mixed	SNNP	HEP	640	32.80%
18	Birhanu et al. [37]	2013	CS	Oromiya	HEP	379	51.7
19	Sinki et al.	2020	Mixed	Oromiya	HEP	604	92.40%
20	Gebretsadik et al. [34]	2019	CS	SNNP	HEP	2279	46.47%
21	Tesfau et al. [35]	2020	CS	Tigray	HEP	705	24.10%
22	Zeleke et al. [38]	2019	CS	Oromiya	HEP	534	41.80%

AA = Addis Ababa; CS = Cross-Sectional; HEP = Health Extension Program; SNNP = Southern Nation Nationalities and peoples; UHEP = Urban Health Extension Program

### Magnitude of health extension services utilization

The prevalence of health extension services utilization in individual studies ranged from 9.3 to 92.4% [27, 39]. The eight included studies were from the Oromiya region, which were conducted at different periods of time, showed that the magnitude of health extension service utilization was 39%, 73.1%, 9.3%, 14.2%, 72.8%, 51.7%, 92.4% and 41.8% [4, 7, 16, 23, 27, 37–39]. The six included studies were from the SNNP region, which were conducted at different periods of time, showed that the magnitude of health extension service utilization was 19%, 69.82%, 61.7%, 12.4%, 32.8% and 46.47% [17, 22, 25, 31, 32, 34]. The four included studies were from Amhara region and were conducted at different periods of time, showed that the magnitude of health extension service utilization was 59.5%, 78.5%, 15.13% and 14.8% [8, 21, 33, 36]. Four different studies were from Addis Ababa, Tigray, Somali and nationwide showed that the magnitude to be 86%, 24.1%, 20.3% and 19.18% respectively [3, 24, 26, 35].

From the 22 included studies in the systematic review, 14 studies reported outcomes that are not reported in any other included studies, only eight studies [3, 4, 7, 8, 21, 25, 38, 39] which reported similar outcome the proportion of health extension service utilization were included in the quantitative synthesis (meta-analysis).

**Fig. 2:**
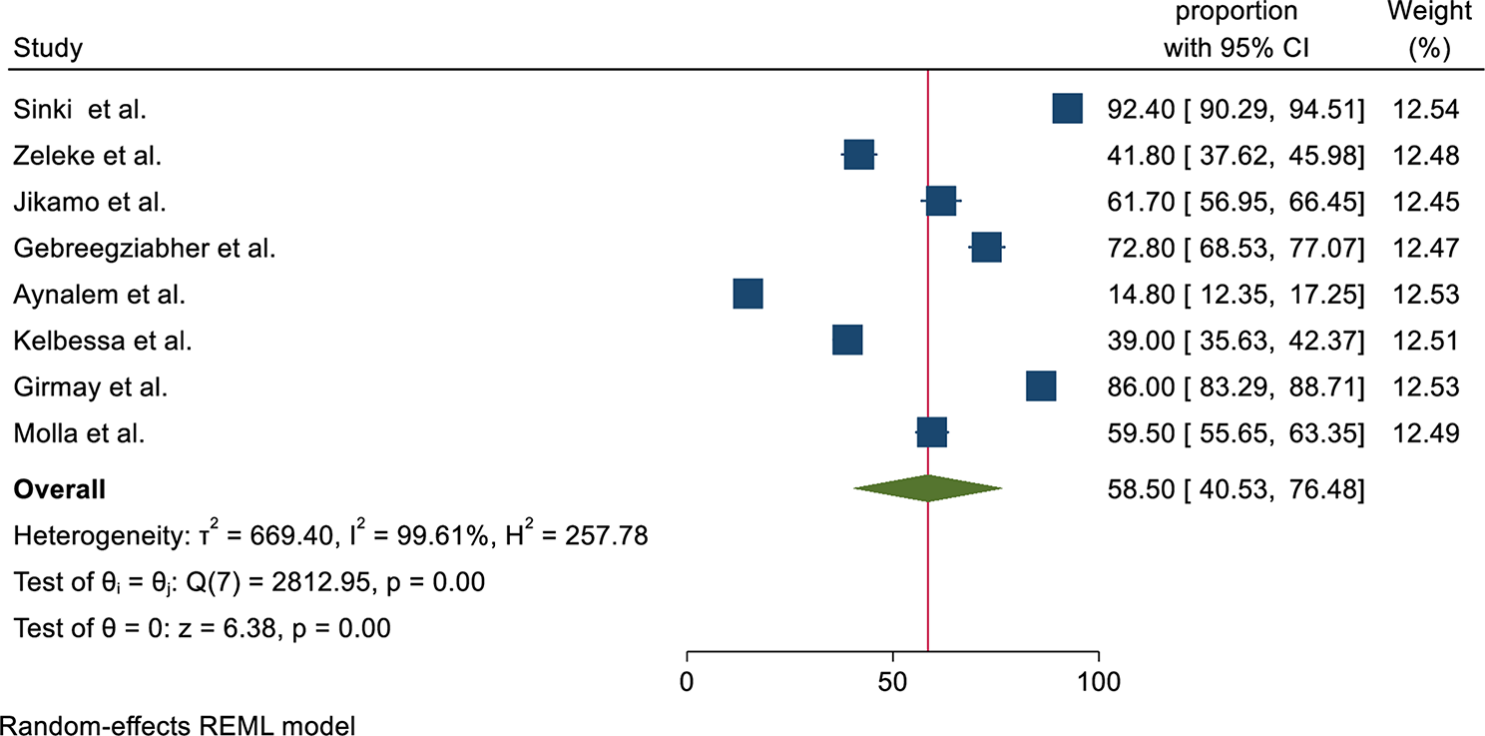
Forest plot of the pooled magnitude of health extension service utilization

The estimated overall magnitude of health extension services utilization is presented in a forest plot (Fig. 2). The overall pooled magnitude of health extension service utilization was 58.5% (95% CI: 40.53, 76.48%). Based on the tau square (between study variance), tau^2^ = 669.40 & I^2^ = 99.61% with p value < 0.01 which indicates there is statistically significant heterogeneity among studies.

### Subgroup analysis

Subgroup analysis was done based on the study design and publication year. Based on this, the magnitude of health extension service utilization was found to be 56.60% and 60.42% in cross-sectional and mixed study designs, respectively. On the other hand, the magnitude of health extension service utilization was 55.87% and 59.38% in studies conducted before and after 2018, respectively (Table 3).

**Table 3:** The pooled proportion of health extension service utilization, 95% CI and heterogeneity estimate with a p-value for the subgroup analysis

Variables	Characteristics	No of studies	Pooled proportion	95% CI	Weight	I^2^(*P*-value)
By study design	Cross sectional	4	56.60%	(35.39, 77.82)	50.01	99.34(0.001)
	Mixed	4	60.42%	(28.07, 92.77)	49.99	99.78(0.001)
	Overall	8	58.5%	(40.53, 76.48)	100	99.61(0.001)
By publication year	Before 2018	2	55.87%	(22.75, 89.00)	24.98	99.33(0.001)
	After 2018	6	59.38%	(36.42, 82.33)	75.02	99.71(0.001)
	Overall	8	58.5%	(40.53, 76.48)	100	99.61(0.001)

### Publication bias

**Fig. 3:**
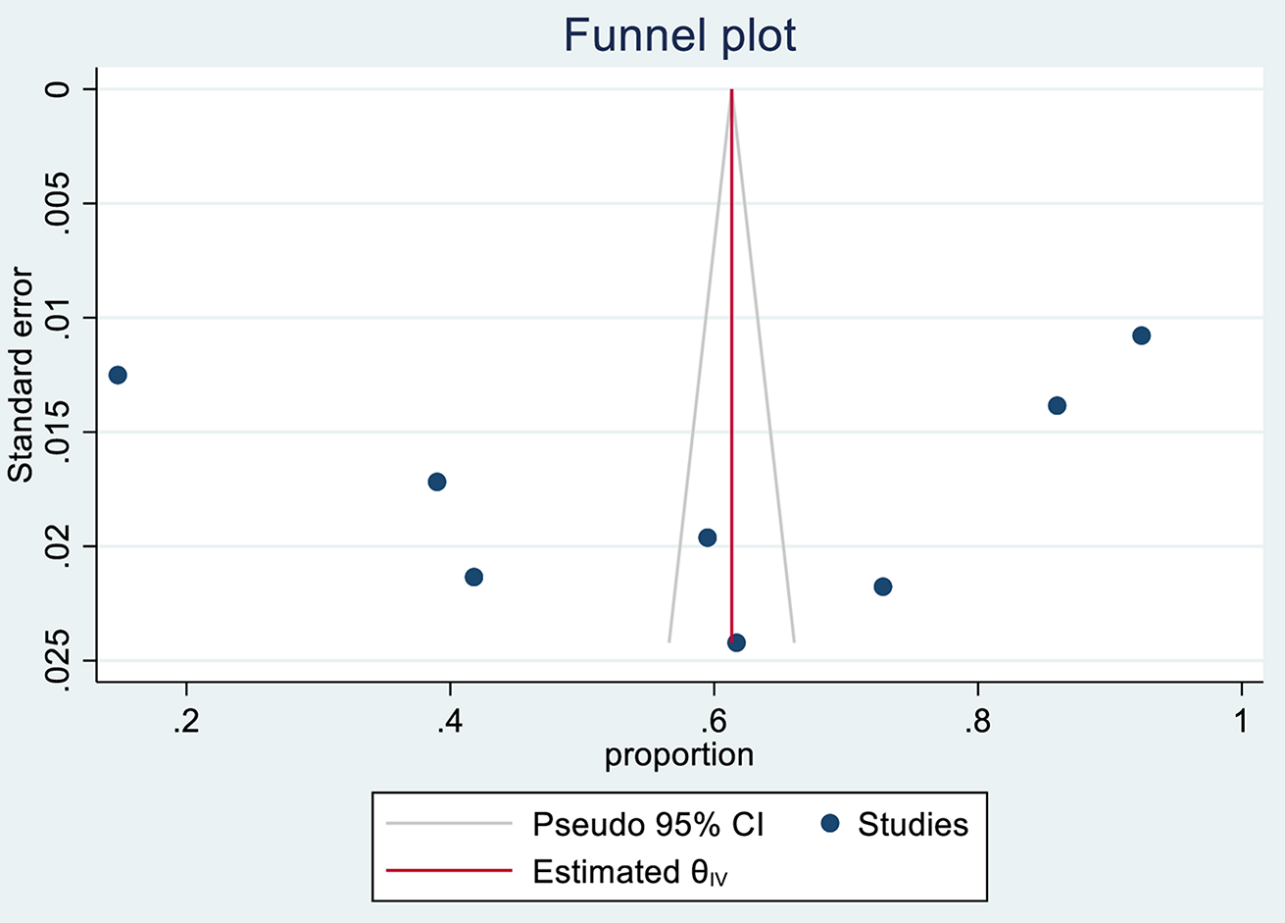
A funnel plot for publication bias assessment

The publication bias was assessed by using a funnel plot (subjectively), and Egger’s and Begg’s tests (objectively). In this study, a funnel plot showed a symmetrical distribution (Fig. 3). Eggers and Begg’s tests also showed no evidence of publication bias at the 0.05 significance level, with a P-value of 0.8155 and 1.00, respectively.

### Sensitivity analysis

**Fig. 4:**
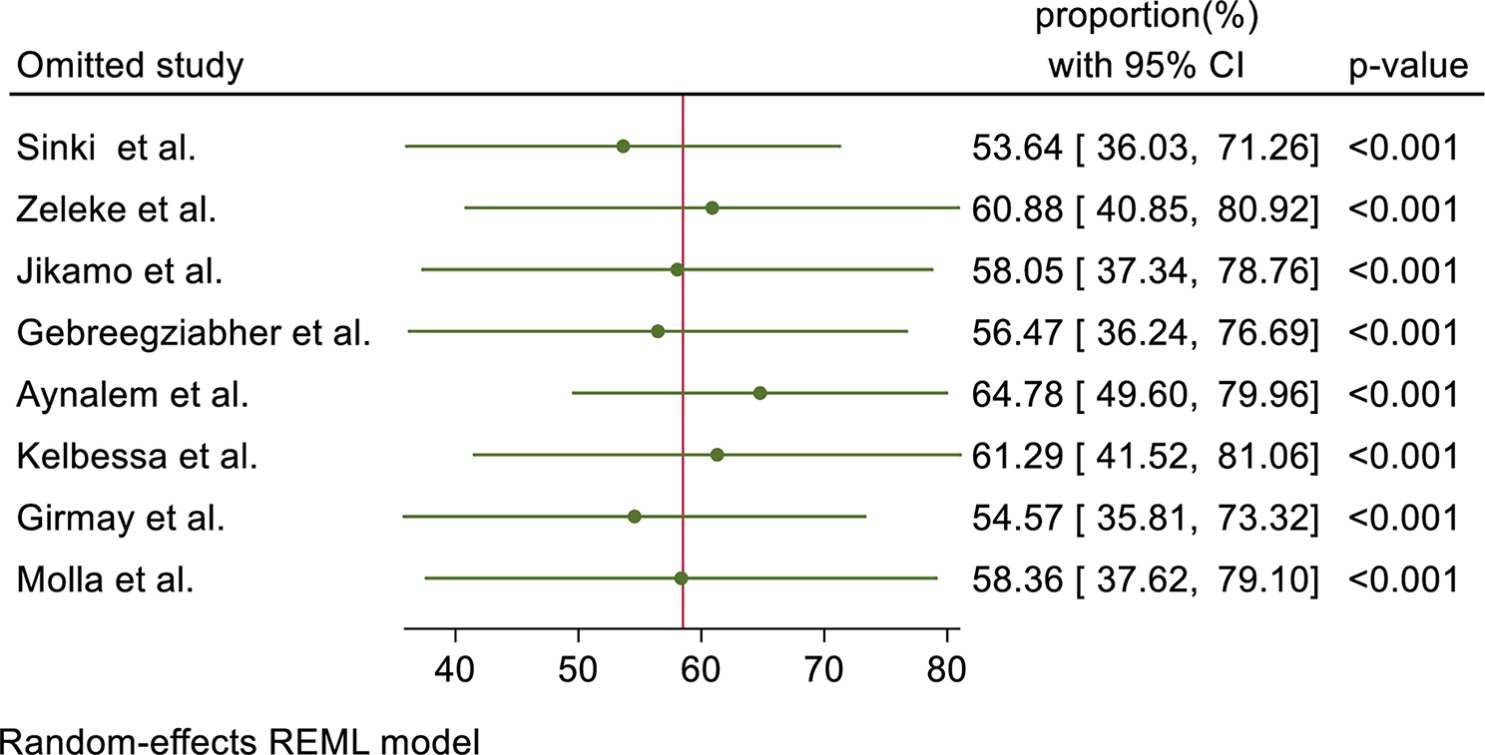
Results of leave-one-out method in sensitivity analysis for pooled proportion of health extension service utilization

The meta-leave-out sensitivity analysis was done to estimate the effect of each study on the pooled magnitude of health extension service utilization by eliminating each study step by step. The result showed that no studies were found to be outside the confidence interval of the pooled proportion of health extension service utilization. Therefore, it showed that all studies had nearly equal influence on the overall pooled proportion of health extension service utilization by excluding the leave-out study from meta-analysis (Fig. 4).

## Discussion

This systematic review and meta-analysis was aimed at estimating the pooled magnitude of health extension service utilization in Ethiopia. The emphasis of this review was to assess the pooled magnitude of health extension service utilization for a better understanding of service delivery and provision at the grassroots level.

The community health extension services are crucial for marginalized groups who face significant barriers to healthcare, particularly in low- and middle-income countries [40–44], and Community Health Workers (CHWs) are one of the cornerstones of comprehensive PHC by providing basic health services and contributing to achieving the key principles of community health and PHC: equity, responding to local health needs, community involvement, and inter-sectorial collaboration [45, 46].

This review revealed that, from the included eight studies, the pooled magnitude of health extension service utilization was found to be 58.5% (95% CI: 40.53, 76.48%). The result of this review was in agreement with a finding from the Ethiopian Demographic and Health Survey (EDHS) of antenatal care (ANC) utilization, which was 62.8% [47], and postnatal care (PNC) utilization of 47% [48]. It was also consistent with findings documented in a study considering child health service delivery by female community health volunteers in Nepal, with a reported child health service provision of 62.6% [49]. Besides, the finding of this review was also comparable with the study conducted in India; the magnitude of community healthcare workers visit was 46.1% [50], and the national utilization of ANC by community health workers in India was 48.9% [51]. Furthermore, it was consistent with studies conducted in western Kenya on the contribution of community health workers for maternal health services (delivery 48% and ANC 66%) [52].

However, the finding of this review was higher than the 2016 EDHS report on institutional delivery service, 26% [20]. The possible justification might be due to the difference in the outcome variable where this review focused on HEP of multiple packages rather than a single service as the only institutional delivery service. It was also higher than a study conducted in community health workers contribution on glycemic control in lower income countries, 21% [53]. The possible explanation for the variation might be due to the methodological differences in which our review included cross-sectional studies rather than a randomized control trial on a glycemic control contribution review of lower income countries. Additionally, the finding of this review was also higher than a study done in Uganda, 27.3% [54]. The possible explanation for this variation might be attributed to the sample size of the study in Uganda, and it was focused on the CHWs contribution from the overall utilization of Integrated Community Case Management (ICCM) services that might be provided by other stakeholders in the study area. Furthermore, this study finding was also higher than a review considered health service utilization in Brazil, 71% [55]. The reason for the variation might be due to the definition of the outcome variable, in which the previous study focused on the overall utilization of health services by all healthcare providers, whereas this review emphasized the utilization of health extension services. The health systems, policies, and government structure might also be the reason for the difference.

The result of this review was lower than a study conducted on the long acting reversible contraception contribution by CHWs in Rwanda, 79% [56] and modern health service utilization in Southern Ethiopia, 77.2% [57]. The possible reason for the variation might be attributed to our study was the pooled result from different studies.

The subgroup analysis of this systematic review and meta-analysis showed that there was no significant difference in health extension service utilization by study design and publication year.

### Policy implications

The result we presented showed that interventions should be taken to increase the utilization of health extension services in Ethiopia. The policymakers might use the finding of this study as an input for developing different health extension service utilization improvement strategies.

## Limitation

The number of studies included in the meta-analysis was small, which may affect the result of the pooled magnitude of health extension service utilization by affecting the precision. We can’t include all the studies in the meta-analysis due to reporting of different outcomes in relation to the HEP.

## Conclusion

This systematic review and meta-analysis reported that health extension service utilization was low compared to the national recommendation. Therefore, the government and policymakers should come up with different mechanisms, including a wide variety of health extension service utilization strategies, so as to achieve universal health coverage through primary health care. Further meta-analysis should also be recommended to identify the associated factors.

### Electronic supplementary material

Below is the link to the electronic supplementary material.

Supplementary Material 1

## Data Availability

All data relevant to the study are included in the article or uploaded as supplemental information.

## Abbreviations

CHWs: Community Health Workers
HEWs: Health Extension Workers
HEP: Health Extension Program
HES: Health extension service
UHC: Universal Health Coverage
