# Supplementary material for: Health extension service utilization in Ethiopia: systematic review and meta-analysis
Source: BMC Health Serv Res. 2024 Apr 26;24:537. doi: 10.1186/s12913-024-11038-4 (PMC11046976; doi:10.1186/s12913-024-11038-4)
Supplement: Supplementary file 1 — Supplementary Material 1 [file 12913_2024_11038_MOESM1_ESM.docx]

Summary of search terms for health extension service utilization

| Databases | Search terms |
| --- | --- |
| PubMed | ("utilization review/legislation and jurisprudence"[MeSH Terms] OR "utilization review/organization and administration"[MeSH Terms] OR "utilization review/standards"[MeSH Terms] OR "utilization review/trends"[MeSH Terms] OR ("statistics and numerical data"[MeSH Subheading] OR ("statistics"[All Fields] AND "numerical"[All Fields] AND "data"[All Fields]) OR "statistics and numerical data"[All Fields] OR "utilization"[All Fields] OR "utilisation"[All Fields] OR "utilisations"[All Fields] OR "utilise"[All Fields] OR "utilised"[All Fields] OR "utilises"[All Fields] OR "utilising"[All Fields] OR "utilities"[All Fields] OR "utility"[All Fields] OR "utilizations"[All Fields] OR "utilize"[All Fields] OR "utilized"[All Fields] OR "utilizer"[All Fields] OR "utilizers"[All Fields] OR "utilizes"[All Fields] OR "utilizing"[All Fields]) OR ("uptake"[All Fields] OR "uptakes"[All Fields] OR "uptaking"[All Fields]) OR ("usage"[All Fields] OR "usages"[All Fields])) AND ("community health services/legislation and jurisprudence"[MeSH Terms] OR "community health services/standards"[MeSH Terms] OR "community health services/supply and distribution"[MeSH Terms] OR "community health services/trends"[MeSH Terms] OR (("health"[MeSH Terms] OR "health"[All Fields] OR "health s"[All Fields] OR "healthful"[All Fields] OR "healthfulness"[All Fields] OR "healths"[All Fields]) AND ("extensibilities"[All Fields] OR "extensibility"[All Fields] OR "extensible"[All Fields] OR "extension"[All Fields] OR "extensions"[All Fields]) AND ("occupational groups"[MeSH Terms] OR ("occupational"[All Fields] AND "groups"[All Fields]) OR "occupational groups"[All Fields] OR "worker"[All Fields] OR "workers"[All Fields] OR "worker s"[All Fields]))) AND ("service"[All Fields] OR "service s"[All Fields] OR "serviced"[All Fields] OR "services"[All Fields] OR "services s"[All Fields] OR "servicing"[All Fields] OR ("package"[All Fields] OR "packages"[All Fields] OR "product packaging"[MeSH Terms] OR ("product"[All Fields] AND "packaging"[All Fields]) OR "product packaging"[All Fields] OR "packaged"[All Fields] OR "packaging"[All Fields] OR "drug packaging"[MeSH Terms] OR ("drug"[All Fields] AND "packaging"[All Fields]) OR "drug packaging"[All Fields] OR "packagings"[All Fields])) AND ("ethiopia/epidemiology"[MeSH Terms] OR ("ethiopia"[MeSH Terms] OR "ethiopia"[All Fields] OR "ethiopia s"[All Fields]) OR ("ethiopian people"[Supplementary Concept] OR "ethiopian people"[All Fields] OR "ethiopians"[All Fields] OR "ethiopian"[All Fields])) |
| Cochrane Library | (service):ti,ab,kw OR (package):ti,ab,kw (Word variations have been searched) AND (Utilization):ti,ab,kw OR (Uptake):ti,ab,kw OR (Usage):ti,ab,kw (Word variations have been searched) AND (Health extension worker):ti,ab,kw OR (Community health worker):ti,ab,kw (Word variations have been searched) AND (Ethiopia):ti,ab,kw OR (Ethiopian):ti,ab,kw (Word variations have been searched) |
| African journals online | ((Service) OR (package)) AND ((Utilization) OR (Uptake) OR (Usage)) AND ((Health extension worker) OR (Community health worker)) AND ((Ethiopia) OR (Ethiopian)) |
